# The reliability, validity and usefulness of the 30–15 intermittent fitness test for cardiorespiratory fitness assessment in military personnel

**DOI:** 10.1038/s41598-022-20315-3

**Published:** 2022-09-27

**Authors:** Armin H. Paravlic, Bostjan Simunic, Rado Pisot, Samo Rauter, Stanko Stuhec, Janez Vodicar

**Affiliations:** 1 Faculty of Sport, University of Ljubljana, Ljubljana, Slovenia; 2 Science and Research Centre Koper, Institute for Kinesiology Research, Koper, Slovenia; 3 Faculty of Sports Studies, Masaryk University, Brno, Czechia

**Keywords:** Physiology, Health occupations

## Abstract

The objectives of this study were to investigate the reliability, validity, and usefulness of the 30–15 intermittent fitness test (30–15_IFT_) in soldiers. The 34 infantry members of the Slovenian armed forces were recruited as participants. Participants performed the continuous incremental treadmill test (TR), a 2-mile run (2_MR_) test, and two 30–15_IFT_ tests. Additionally, participants were divided into a highest-scoring group (HSG) and a lowest-scoring group (LSG) based on their scores on the Army Physical Fitness Test. A *very high* reliability ratings were observed for 30–15_IFT_ measures, as follows: end-running speed (ERS) ERS_IFT_ (ICC = 0.971)_,_ maximal heart rate (HR_max_) HR_maxIFT_ (IC = 0.960)_,_ and maximal relative oxygen consumption (VO_2max_) VO_2max-IFT_ (ICC = 0.975)_._ Although 30–15_IFT_ measures demonstrated high correlations (r = 0.695–0.930) to the same measures of TR test, ERS, HR_max_ and VO_2max_ were higher in the 30–15_IFT_ (p > 0.05)_._ Furthermore, ERS_IFT_ and predicted VO_2maxIFT_ were higher in HSG compared to LSG, whereas HR_max_ did not differ. The results of this study show that the 30–15_IFT_ test is a reliable, valid and useful tool for assessing cardiorespiratory fitness in the armed forces. Moreover, the ERS and predicted VO_2_max values derived from the 30–15_IFT_ could be considered more sensitive markers of combat readiness than the parameters derived from the TR and 2_MR_ tests.

Trial registration number: NCT05218798.

## Introduction

Everyday military work involves a variety of movement patterns, including walking, running, jumping, pushing, pulling, lifting, throwing, kicking, dragging, crawling, shooting a firearm, carrying a heavy backpack and/or other equipment, all of which put a lot of strain on the soldier's body^1^. Soldiers must be very physically fit in order to carry out later occupational responsibilities with ease and increased efficiency^1,2^. Similar to other physically demanding occupations such as firefighting, and/or law enforcement, muscular strength, strength endurance, and cardiorespiratory fitness (CRF) have been shown to be one of the most important components of a soldier's physical fitness profile^3–5^. To test these attributes, there are many individual tests and test batteries, of which the Army Physical Fitness Test (APFT) is the most widely used test battery for military personnel due to its simplicity and ease of administration^1,3^. The APFT, which consists of sit-ups, push-ups and a 2-mile run (2_MR_), was introduced in 1980 for members of the US Army. It is design to measure upper body and core muscles strength endurance and CRF^1,3^. While muscle strength and core muscle strength endurance are relatively easy to measure, assessing CRF in this special population appears to be more challenging due to the specifics of military operations, which are complex and often unpredictable^1,6,7^. CRF testing is routinely performed by most armed and tactical forces around the world as part of their recruitment process for new members or simply as an annual examination of their personnel^1,7^. Indeed, direct measurement of CRF during an incremental run on a treadmill (TR) is considered the "gold standard" for estimating maximal relative oxygen uptake (VO_2max_)^8^. However, because of its high cost, complex measurement procedures, and inability of group testing, this method is not routinely used to examine CRF in soldiers^1,3^. In addition to the 2_MR_, several other field tests such as the 12-mile run, the 3000-m run, the 2400-m run, and the 1-mile run are used for CRF evaluation of military personnel^3,9^. Later tests are usually conducted outdoors and were found to have questionable validity ranging from low to high^10,11^. Therefore, weather, climate, and terrain can influence the results and may limit the maximum and reliable performance. Although standardized, the most commonly used tests, such as the 2_MR_, have been shown to be difficult for individuals because the pacing strategy is internally controlled^7^. As such, it does not reflect the real situation on the battlefield, where most activities are externally driven by the environment and the enemy. Running patterns on the battlefield are characterized by intermittent, high-intensity shuttle runs with constant changes of direction combined with forward and backward running and turning, while unexpected interruptions of running are common when soldiers are exposed to open fire from the enemy. Furthermore, a high standard of strength and conditioning planning and programming required for military personnel cannot be met with such a test because the final result is expressed as the total time required to run a given distance without any other information needed for training prescription and necessary optimization. Over the past two decades, an intermittent shuttle run testing with 30–15 Intermittent Fitness Test (30–15_IFT_) has been successfully implemented for CRF testing in various populations^12–14^. 30–15_IFT_ is an incremental test consisting of 30-s shuttle runs interspersed with 15-s active recovery periods. In addition, it has been demonstrated to accurately predict the VO_2max_ values in both elite and young male handball, soccer and hockey players^12,14^, respectively. This suggests that it can be utilized for both CRF assessment and individual training prescription^12,15^. Other useful measurements, such as maximal heart rate (HR_max_) and end-running speed (ERS), can be generated from 30–15_IFT_ in addition to predicted VO_2max_. According to a recent study^14^ elite handball players' ERS and VO_2max_ values were significantly greater when determined from the 30–15IFT test than when produced from the TR test. As a result, prescribing an aerobic training intensity based on ERS may vary significantly (i.e., up to 3.2 km/h or 19%)^14^ and harm athletes' CRF development.

The recent reports of the International Congress on Soldiers' Physical Performance emphasize that the two most important priorities in military research and practice are the monitoring of physiological condition and the study of physical demands in operational environments, which need to be improved^16^. Considering all this, we believe that the 30–15_IFT_ could be a suitable tool for measuring CRF of military personnel.

To date, no study has examined the reliability and validity of the 30–15_IFT_ compared to a standard continuous incremental running test and/or a 2_MR_ in infantry members. From a practical perspective, it would be of great interest to Slovenian Armed Forces (SAF) personnel to provide their strength and conditioning coaches with a valuable measure of CRF to determine and monitor the readiness of SAF infantry members. We hypothesized that the 30–15_IFT_ will prove to be a highly reliable and valid measure of CRF while other parameters such as HR_max_ and ERS_IFT_ will be valuable indicators of IFT data for prescribing, monitoring, and optimizing high-intensity interval training in this population.

## Methods

### Study design

This is a randomized cross-over study using a within-subjects test–retest design. To test the current hypothesis, a TR test, a 2_MR_ test, and two 30–15_IFT_ were performed in the population of SAF infantry members. The present study was conducted over a maximum period of 2 weeks per participant. Participants were instructed to visit the laboratory four times at least 72 h apart. At the first visit, they were familiarized with the experimental procedures and asked to sign a written informed consent form to participate in the study. Then, participants were randomly assigned to four experimental conditions (i.e., TR, 2_MR_ and two 30–15_IFT_ running tests).

Additionally, participants were divided into two groups based on their score on the APFT (score ranging from 1 to 5). Thus, the highest-scoring group (HSG) is defined as an APFT score of 5; and the lowest-scoring group (LSG) is defined as an APFT score of ≤ 2.

In the conceptualization phase of the study, we conducted an a priori power analysis for the estimated intraclass correlation coefficient (ICC). Based on previous studies with similar aim we expected to find high to nearly perfect ICC for the reliability of 30–15_IFT_ test^15,17^. Therefore, with the research power of 0.90 and two-tiled α = 0.05, a minimum sample size of 9 participants showed to be sufficient to detect a value of ≥ 0.80 for the ICC.

### Participants

Thirty-four SAF infantry members (males, N = 27) were recruited for the proposed study. Inclusion criteria were: age 18–40 years, both sexes, SAF members who are performing regular military tasks on daily basis, with no history of injuries in the last 6 months prior to recruitment, have not reported any musculoskeletal pain, with no history of chronic musculoskeletal, metabolic, pulmonary, neurological and cardiovascular diseases. Not satisfying an inclusion criterion, such as being under 18 or older than 40, having a musculoskeletal injury or illness that is either chronic or recent, was established as an exclusion criterion. Participants were instructed to avoid any strenuous physical activity for at least three days prior to the start of the first testing session and during the course of study, which was monitored by their superiors. They were requested to refrain from consuming ergogenic substances prior to the tests. Before the initial assessment on each testing day, a brief meeting was held to explain the study protocol in detail.

### Experimental procedures

The experimental procedures were thoroughly explained in the recently published protocol of the current study^18^. In brief, all tests were performed at the facilities at the Faculty of Sport (intentionally deleted) between 8 a.m. and 1 p.m. The actual arrival of participants to the testing location was pre-planned and coordinated with the commanding officers. Each daily testing group had 6–8 participants. The timeline of testing procedures is explained in detail in Table 1. The study was approved by the Slovenian National Medical Ethics Committee, Ministry of Health (Ljubljana, Slovenia; reference number: 0120-495/2021/6) and conducted in accordance with the 1964 Declaration of Helsinki and its subsequent amendments.

**Table 1:** Timeline of the study protocol.

Activity/measurements	Time (min)
Body height, mass and body composition	5
Warm-up routine—upright cycling 5 min (1 week per kg of body weight, 70 rpm) Part I	5–10
Dynamic stretching Part II	10–15
Specific warm-up (running 8 km/h) Part III	15–25
Actual aerobic test (TR, 2_RM_ or 30–15_IFT 1 and/or 2_)	25–50

*TR* treadmill run test, *2*_*RM*_ 2-mile running test, *30–15*_*IFT*_ Intermittent Fitness Test, *rpm* revolutions per minute.

### Body composition assessment

Body mass and height were measured using a stadiometer and scale anthropometer (GPM, Model 101, Zurich, Switzerland) to the nearest 0.1 cm, while body mass was assessed with multifrequency bioelectrical impedance (InBody 720: Biospace, Tokyo, Japan) to the nearest of 0.05 kg. Additionally, muscle mass (MM%) and fat mass (FM%) were calculated using manufacturer’s algorithm.

### Physiological testing

A portable metabolic gas analyzer (K5, COSMED, Italy) was used to obtain physiological parameters. Simultaneously, a heart rate was measured by heart rate monitoring belt (Garmin Edge 830 Pack, Kansas, United States). The data were recorded in 5-s interval and automatically analyzed by using the original Polar software.

### Continuous incremental running treadmill test

After 5 min of baseline measurements, while standing on the treadmill (HP Cosmos, Germany), the participants warmed-up at 8 km/h run and constant gradient of 1% inclination, as previously suggested^19^. Then, test was executed by running until volitional exhaustion where running speed was increased progressively by 2 km/h per minute. The achievement of VO_2_max was identified as the plateauing of VO_2_ (< 2.1 ml/kg/min decrease) despite an increase in workload^20^. If the above-stated criterion was not fulfilled, the participants were asked to perform a further constant-speed test equal or higher than the highest speed achieved at the end of the incremental test, as recommended^21^. Throughout the test, respiratory gases were continuously measured breath-by-breath and reduced to 10-s averages^22^. ERS was determined as the minimal running velocity that elicited VO_2_max over a period of 30 s.

### Continuous 2-mile run test

The 2_MR_ was used as a continuous field test and was performed on a 400-m synthetic athletic track with the supervision of the research team. Participants were required to complete the 2_MR_ course without any physical help in the shortest time possible. The heart rate at the end of the 2_MR_ was considered the HR_max_ achieved in the test.

### 30–15 intermittent fitness test

30–15_IFT_ test consists of 30-s shuttle runs interspersed with 15-s active recovery periods. Running speed was set at 8 km/h for the first 30-s run and increased by 0.5 km/h in each 30-s phase thereafter, while the participants were required to run back and forth between two lines 40 m apart at the predetermined pace determined by a prerecorded beep like elswhere^14,18^. The speed of the last successfully completed stage was recorded as the test result, that is ERS_IFT_^15^, while the VO_2max_ was calculated by the previously proposed formula^15^.

### Statistical analysis

All data are be presented as mean ± SD with 95% confidence interval limits [95% CI]. All statistical analysis was done using the SPSS statistical software (version 27.0, IBM Inc, Chicago, USA). Descriptive statistics was used to summarize demographic characteristics of participants and outcomes. Normality od the data was confirmed by Shapiro–Wilk test, while the homogeneity of variances of normally distributed variables was tested by Levene’s test.

Differences in key physiological outcomes between the different CRF tests (TR vs. 2_MR_ vs. 30–15_IFT_) were assessed by 1-way Analysis of variance (ANOVA). *Cohens’ d* (ES) was used to assess magnitude of difference between tests and was interpreted as: trivial: < 0.20, small: 0.20–0.50, moderate: 0.50–0.80, or large: > 0.80^23^. The relative reliability of all dependent variables between two 30–15_IFT_ trials was estimated using the ICC, two-way random effects model (consistency type). ICC values were classified as: very high: > 0.90, high: 0.70–0.89, and moderate: 0.50–0.69. The following criteria was used to declare good reliability: CV < 5% and ICC > 0.69^17^. In addition, the standard error of the estimate (SEM) and the coefficient of variation (CV) were calculated as measures of absolute reliability, indicating the within-subject variation, as previously proposed^24^. To test the usefulness of the IFT, the spreadsheet provided by Hopkins was used^25^. Usefulness was determined by comparing the smallest worthwhile change (SWC) with the typical error of measurement (TE) and was interpreted as follows: Marginal: TE > SWC, OK: TE = SWC, and Good: TE < SWC^25^.

Relationship between VO_2_max, HR_max_ and ERS_IFT_, TR and 2_MR_ was assessed using Pearson’s correlation (r). Also, the relationship between VO_2_max obtained from TR, 2_MR_ and 30–15_IFT_ was investigated. The following thresholds of the correlation coefficient were used to assess the magnitude of the relationships analyzed: weak: ≤ 0.35; moderate: 0.36–0.67; and high: ≥ 0.68^26^.

In addition, to determine performance differences between two groups of SAF members, a repeated-measures General Linear Model was used for main parameters estimated from different tests (TR vs. 30–15_IFT_ vs. 2_MR_) as within-subject factor, whereas groups (HSG vs. LSG) was used as between-subject factors. Partial eta squared (η^2^) values of 0.01, 0.06 and 0.14 rated difference as small, moderate and high, respectively^23^. Statistical significance for all analysis conducted was accepted at p ≤ 0.05.

## Results

Shapiro–Wilk’s test confirmed that all data were normally distributed (p > 0.05).

Similar V_IFT_ (test: 17.25 ± 1.75 km/h; re-test: 17.43 ± 1.67 km/h) and VO_2max-IFT_ (test: 49.24 ± 5.94 ml/kg/min; re-test: 49.76 ± 5.66 ml/kg/min) were observed between two 30–15_IFT_ testing trials, while HR_max_ (test: 193.50 ± 8.97 b.p.m.; 190.85 ± 10.01 b.p.m) values differed (Table 2). A very high test–retest reliability (ICC > 0.90; CV% ≤ 1.82) (Table 2).

**Table 2:** Reliability analysis for end-running speed (ERS_IFT_), maximal heart rate (HR_max_) and maximal relative oxygen consumption (VO_2max_) during 30–15 intermittent fitness test (30–15_IFT_).

	ERS_IFT_ (m/s)	HR_maxIFT_ (bpm)	VO_2max_ (mlO_2_/min/kg)
30–15_IFT_ 1st trial	17.25 ± 1.75	193.50 ± 8.97	49.24 ± 5.94
30–15_IFT_ 1st trial	17.43 ± 1.67	190.85 ± 10.01	49.76 ± 5.66
p-value	0.083	**p < 0.001**	0.103
Mean difference [95% CI]	− 0.18 [− 0.38; 0.02]	2.65 [1.35; 3.94]	− 0.52 [− 1.14; 0.11]
Effect size [95% CI]	− 0.31 [− 0.65; 0.04]	0.71 [0.33; 1.09]	− 0.29 [− 0.63; 0.06]
CV% [95% CI]	1.72 [1.11; 2.33]	1.29 [0.91; 1.68]	1.82 [1.15; 2.48]
TE [95% CI]	0.24 [0.20; 0.32]	0.29 [0.23; 0.38]	0.22 [0.18; 0.30]
SWC [95% CI]	0.34 [0.28; 0.45]	1.90 [1.53; 2.50]	1.16 [0.94; 1.53]
ICC [95% CI]	0.971 [0.942; 0.986]	0.960 [0.920; 0.980]	0.975 [0.951; 0.988]
Effect size rating	*Small*	*Moderate*	*Small*
Usefulness rating	Good	Good	Good
Reliability rating	Very high	Very high	Very high

*CV* coefficient of variation, *TE* typical error of measurement, *SWC* smallest worthwhile change, *ICC* intraclass correlation coefficient.

Bold values, statistically significant.

The TE for ERS_IFT_ (TE = 0.24 km/h), HR_max_ (TE = 0.29 bpm) and VO_2_max_IFT_ (TE = 0.22 ml/kg/min) were lower than presumed SWC (ERS_IFT_ = 0.34 km/h; HR_max_ = 1.90 bpm; VO_2_max_IFT_ = 1.16 ml/kg/min) and thus, these measures were rated as “Good” (Table 2).

Significant differences were observed between TR, 30–15_IFT_ and 2_MR_ tests for ERS (high η^2^ = 0.623; p < 0.001), HR_max_ (high η^2^ = 0.158; p < 0.001), and VO_2max_ (high η^2^ = 0.160; p < 0.001) (Table 3).

**Table 3:** Observed results for end-running speed (ERS), maximal heart rate (HR_max_) and maximal relative oxygen consumption VO_2max_ in treadmill running test (TR), 30–15 intermittent fitness (30–15_IFT_) test and 2-mile run (2_MR_) test.

	TR	30–15_IFT_	2_MR_	F_test_ (p value): [η^2^]
ERS	13.65 ± 1.89	17.25 ± 1.75*^©^	11.96 ± 1.57	81.841 (**p <** 0.**001**): [0.623]
HR_max_	184.62 ± 9.30	193.50 ± 8.97*^©^	185.18 ± 10.26	9.271 (**p <** 0.**001**): [0.158]
VO_2max_	44.86 ± 7.37	49.24 ± 5.94*^©^	42.56 ± 5.92	9.426 (**p <** 0.**001**): [0.160]

*Significantly higher than TR.

^©^Significantly higher than 2_MR._

Bold values, statistically significant.

Post hoc analysis showed significant differences between ERS_IFT_ and ERS_TR_ (MD: 3.60 km/h; *large ES* = 3.01; 95% CI [2.21; 3.80]; p < 0.001); ERS_IFT_ and ERS_2MR_ (MD: 5.29 km/h; *large ES* = 7.62; 95% CI [5.75; 9.47[; p < 0.001); ERS_TR_ and ERS_2MR_ (MD: 1.69 km/h; *large ES* = 1.54; 95% CI [1.03; 2.03]; p < 0.001); VO_2_max_IFT_ and VO_2_max_2MR_ (MD: 6.68 ml/kg/min; *large ES* = 1.18; 95%CI [0.73; 1.59]; p < 0.001); HR_maxIFT_ and HR_maxTR_ (MD: 8.89 bpm; *large ES* = 1.37; 95% CI [0.90; 1.84]; p = 0.001); HR_maxIFT_ and HR_max2MR_ (MD: 8.32 bpm; *large ES* = 1.40; 95% CI [0.92; 1.87]; p = 0.001), respectively. Furthermore, a high correlation was observed between ERS_IFT_ and VO_2_max_IFT_ (r = 0.930; p < 0.001), ERS_TR_ (r = 0.786; p < 0.001), VO_2_max_TR_ (r = 0.695; p < 0.001), ERS_2MR_ (r = 0.919; p < 0.001), VO_2_max_2MR_ (*moderate* r = 0.621; p < 0.001) and running time on the 2_MR_ (r = − 0.916; p < 0.001) (Table 4). Also, HR_maxIFT_ showed high correlation with HR_maxTR_ (r = 0.751; p < 0.001) and HR_max2MR_ (r = 0.816; p < 0.001) (Table 4).

**Table 4:** Pearson’s correlation coefficient (r) between end-running speed (ERS), maximal heart rate (HR_max_) and maximal relative oxygen consumption VO_2max_ parameters obtained during a treadmill running test (TR), 30–15 intermittent fitness (30–15_IFT_) test and 2-mile run (2_MR_) test.

	HR_maxIFT_	VO_2maxIFT_	ERS_TR_	HR_maxTR_	VO_2maxTR_	ERS_2MR_	HR_max2MR_	VO_2max2MR_	RT_2MR_
ERS_IFT_ (km/h)	0.194	0.930**	0.786**	0.120	0.695**	0.919**	0.112	0.621**	− 0.916**
HR_maxIFT_ (bpm)		0.084	0.163	0.751**	0.134	0.084	0.816**	0.133	− 0.078
VO_2maxIFT_ (ml/kg/min)			0.754**	0.080	0.664**	0.875**	0.053	0.543**	− 0.878**
ERS_TR_ (km/h)				0.268	0.757**	0.814**	0.124	0.592**	− 0.833**
HR_maxTR_ (bpm)					0.259	0.016	0.789**	0.163	− 0.033
VO_2maxTR_ (ml/kg/min)						0.712**	0.083	0.796**	− 0.714**
ERS_2MR_ (km/h)							0.057	0.620**	− 0.985**
HR_max2MR_ (bpm)								0.104	− 0.053
VO_2max2MR_ (ml/kg/min)									− 0.614**

*bpm* beats per minute.

**Correlation is statistically significant at the p ≤ 0.01 level (2-tailed), denoted by bold print.

HSG ERS_IFT_ (mean difference [MD]: 2.57 km/h; 95% CI [1.31, 3.83]; p = 0.001), and predicted VO_2_max_IFT_ (MD: 8.79 ml/kg/min; 95% CI [4.83, 12.74]; p < 0.001) were higher than in LSG, whereas HR_max_ did not differ (p = 0.333) (Table 5).

**Table 5:** Comparisons between highest scoring group (HSG) and lowest scoring group (LSG) on Army Physical Fitness test in end-running speed (ERS), maximal heart rate (HR_max_) and maximal relative oxygen consumption VO2max parameters obtained during a treadmill running test (TR), 30–15 intermittent fitness (30–15_IFT_) test and 2-mile run (2_MR_) test.

Parameters	HSG (n = 15)	LSG (n = 8)	Mean difference	T/F value (p value) [η^2^]	ES [95% CI]
**Basic demographic characteristics**					
Age (years)	35.27 ± 7.95	31.44 ± 5.55	3.82	1.264 (0.219)	NA
Sex (males/females)	13/2	8/1	NA	NA	NA
**Body composition**					
Body mass (kg)	79.65 ± 10.94	87.97 ± 14.52	− 8.31	− 1.595 (0.125)	NA
Body mass index (kg/m^2^)	24.84 ± 1.65	27.53 ± 3.86	− 2.69	− 2.387 (**0.026**)	− 1.01 [− 1.87; − 0.12]
Muscle mass (%)	48.46 ± 3.34	44.19 ± 3.34	4.27	3.034 (**0.006**)	1.28 [0.36; 2.17]
Fat mass (%)	13.35 ± 5.25	20.43 ± 6.26	− 7.09	− 2.983 (**0.007**)	− 1.26 [− 2.15; − 0.34]
**Cardiorespiratory fitness related measures**					
ERS_TR_ (km/h)	14.60 ± 1.50	12.89 ± 2.03	1.71	13.974 (**0.001**) [0.388]	1.00 [0.11; 1.87]
ERS_IFT_ (km/h)	18.57 ± 1.32	16.00 ± 1.46	2.57		1.87 [0.86; 2.85]
ERS_2MR_ (km/h)	13.03 ± 1.19	10.91 ± 1.54	2.12		1.60 [0.64; 2.54]
HR_maxTR_ (bpm)	184.33 ± 9.82	187.00 ± 8.80	− 2.67	0.684 (0.417)	NA
HR_maxIFT_ (bpm)	192.20 ± 9.63	195.89 ± 7.27	− 3.69		− NA
HR_max2MR_ (bpm)	183.93 ± 12.17	187.11 ± 8.95	− 3.18		NA
VO_2maxTR_ (ml/kg/min)	49.11 ± 6.11	42.16 ± 6.78	6.95	15.871 (**0.001**) [0.419]	1.09 [0.20; 1.97]
VO_2maxIFT_ (ml/kg/min)	53.76 ± 4.48	44.97 ± 4.60	8.79		1.94 [0.92; 2.93]
VO_2max2MR_ (ml/kg/min)	45.44 ± 5.00	39.94 ± 4.22	5.50		1.16 [0.26; 2.04]
RT_2MR_ (s)	890.87 ± 79.80	1075 ± 151.4	− 184.13	− 3.921 (**0.001**)	− 1.65 [− 2.60; − .68]

*bpm* beats per minute.

Bold values, statistically significant.

The differences observed between HSG and LSG were greater when values derived from IFT were considered, compared to TR and 2MR for both ERS and predicted VO_2_max (Table 5). Furthermore, HSG showed to have higher muscle mass percentage (MD: 4.27%; 95% CI [1.35, 7.19]; p = 0.006), lower fat mass (MD: − 7.09%; 95% CI [− 12.01, − 2.16]; p = 0.007), and body mass index (MD: − 2.69 kg/m^2^; 95% CI [− 5.03, − 0.35]; p = 0.026) compared to LSG (Table 5).

## Discussion

The purpose of this study was to examine the reliability, criterion validity and usefulness of the 30–15_IFT_ within SAF infantry members. In agreement with previous research on the topic, our results suggest that ERS_IFT_ obtained during the 30–15_IFT_ provides a reliable, valid and useful method of assessing soldier’s CRF. A *very high* reliability ratings (ICC = 0.971–0.975) were observed for ERS_IFT,_ HR_maxIFT_ and VO_2_max_IFT_, with *small* (ERS_IFT_ and VO_2_max_IFT_) to *moderate* (HR_maxIFT_) test–retest differences. Although outcome measures obtained during the 30–15_IFT_ demonstrated high correlations (r = 0.695–0.930) to the same measures obtained during the gold-standard continuous TR, ERS, HR_max_ and VO_2_max were higher in the 30–15_IFT_ (Table 3). This is in good agreement with previous findings^14,27^. Also, by showing good rating for all 30–15_IFT_ outcome measures, our results confirmed the usefulness of the 30–15_IFT_ for testing soldier’s CRF.

The 30–15_IFT_ has gained popularity among practitioners and researchers in the field of exercise science. Before it can be used as a field instrument to routinely monitor CRF in infantry members, it is important to investigate its physiometric properties such as reliability and criterion validity. Our data demonstrate both relative (i.e., ICC) and absolute (i.e., CV) reliability of all outcome measures collected with the 30–15_IFT_ in this specific population. The comparable findings were observed elsewhere^27–29^. The most recent systematic review aimed to investigate the test–retest reliability of the 30–15_IFT_ found that ICC values for ERS ranged from 0.80 to 0.99, whereas ICC values for HR_max_ ranged from 0.90 to 0.97^29^. In addition, the authors found that the CV values for ERS ranged from 1.5 to 6% (1.72% in the present study), whereas the CV values for HR_max_ ranged from 0.6 to 4.8%^29^ (1.29% in the presents study). The observed variation in ICCs and CVs between later studies might be prescribed to different population studied and a level of familiarization with the testing battery implemented^29,30^. The population studied comprised of athletes competing in different sports, and thus 30–15_IFT_ test for some sports might be specific compared to others. Also, it is well known that an individual may become more proficient in each test with increased experience^30^. However, a *small* difference observed between test–retest trials for ERS (MD: 0.18 km/h), with SWC of 0.34 km/h, which is less than 1 stage (0.5 km/h), suggests absence of a learning effect in our sample. Furthermore, our results have shown that high reliability can be achieved with a single familiarization session and detailed instructions on how to perform the test.

Besides the reliability and the validity, the information on usefulness of the 30–15_IFT_ test will allow sports scientist to make firm conclusion about whether the changes observed in response to strength and conditioning programming intervention are important or not^24^. Our results showed good usefulness rating for ERS_IFT,_ which is in good agreement with previous findings^28,31–33^. For example, for ERS_IFT,_ SWC was found to be *marginal* (SWC = 0.20 km/h, TE = 0.56 km/h), *marginal* (SWC = 0.20 km/h, TE = 0.31 km/h) and *ok* (SWC = 0.34 km/h, TE = 0.32 km/h), in female basketball players^32^, female football players^27^ and male futsal players^31^, respectively. Thus, comparable with results observed among athletic population the 30–15_IFT_ test showed to be useful for testing and monitoring training intervention progress in military personnel. This data suggests that any improvements in performance as small as 0.5 km/h (one stage) can be considered real and meaningful in military personnel.

A comparison of HSG and LSG level soldiers showed differences in 30–15_IFT_. HSG reached greater ERS_IFT_ (MD: 2.57 km/h) and had higher VO_2_max_IFT_ (MD: 8.79 mlO_2_/kg/min). A mean ERS_IFT_ and VO_2_max_IFT_ differences between groups were greater than SWCs (ERS_IFT_ > 0.5 km/h; VO_2_max_IFT_ > 1.16 mlO_2_/kg/min), suggesting that there were meaningful differences between HSG and LSG. Furthermore, HR_max_ is also considered useful for detecting meaningful performance changes in the individual as small as 2 bpm. This confirms previous findings in female football players^27^, and male rugby^34^ and futsal^31^ players. Although this is the first study aimed to investigate reliability, validity and usefulness of 30–15_IFT_ in military personnel, our results are in good agreement with findings observed in athletic population^27,34^. Later studies revealed differences in 30–15_IFT_ test performance between players of different levels, whereas for example a national team female football players achieved for 1.15 km/h greater ERS and had for 2.2 mlO_2_/kg/min greater predicted VO_2max_ then national club players^27^. It is interesting to note that magnitude of differences observed between HSG and LSG were greater when values derived from IFT were considered, compared to TR and 2_MR_ for both ERS and predicted VO_2max_ (Table 3). This suggests that performance parameters obtained during the IFT test, such as ERS and VO_2_max_,_ may be more sensitive markers for distinguishing between better and less prepared soldiers than the same variables derived from TR and 2^MR^ tests. We believe these results can help practitioners working with military personnel to guide and optimize their strength and conditioning programming, especially when it comes to CRF interval training. The ERS obtained with the 30–15_IFT_ showed to be 5.29 km/h higher than ERS obtained from 2_MR_ test. This valuable information suggests that practitioners still using the 2_MR_ test for CRF evaluation in military personnel and looking for the optimal running speed for interval training targets should add 5.29 km/h to the average speed obtained with the 2_MR_ test to achieve the required aerobic speed.

Although the present study has many unique aspects, such as the novelty of the population studied and the direct comparison of the results of three different endurance tests, there are certain limitations in the current investigation that need to be pointed out. Due to the low proportion of female subjects, we were unable to conduct separate reliability analyses that considered the sex of the participants. However, women make up only 7% of the SAF's soldiers, and considering equal right and opportunities, our study is not far from the real situation in the SAF. Further studies aimed at establishing normative values for the 30–15IFT for SAF infantry members and investigating its relationship with occupation-specific test batteries such as the ACFT and/or the Marine Combat Fitness Test are warranted.

## Conclusion

The results of this study show that the 30–15_IFT_ test is a reliable, valid and useful tool for assessing CRF in military personnel. Moreover, the ERS and predicted VO_2_max values derived from the IFT test could be more sensitive markers of combat readiness than the same variables derived from the TR and 2_MR_ tests. Therefore, the 30–15_IFT_ test can be reliably used by researchers and strength and conditioning coaches working with military personnel to test and monitor cardiorespiratory adaptations to training interventions.

## Data Availability

The data that will support the findings of proposed study will be available on reasonable request from the corresponding author.
